# Supplementary material for: Geographical Indication Characteristics of Aroma and Phenolic Acids of the Changping Strawberry
Source: Foods. 2023 Oct 24;12(21):3889. doi: 10.3390/foods12213889 (PMC10650669; doi:10.3390/foods12213889)
Supplement: Supplementary file 1 [file foods-12-03889-s001.zip › foods-2644045-supplementary.pdf]

Table S1. Aroma composition and relative content in strawberries from different groups.

		Relative content (%)					
No.	Compound Name and Class	GI	CP	PG	MY	SY	TZ
	Furanones	2.05	3.11	2.44	3.03	2.15	3.16
1	2-Methyl furan	0.02	0.01	0.01	0.03	0.02	0.01
2	2-Ethyl furan	0.10	0.10	0.14	0.23	0.19	0.14
3	2-Penty lfuran	1.35	1.77	1.84	2.56	1.71	2.46
4	2,5-Dimethyl-4-methoxy-3(2H)-furanone	0.48	1.07	0.37	0.15	0.17	0.38
5	Furaneol	0.11	0.16	0.08	0.06	0.07	0.17
	Lactones	0.35	0.60	0.21	0.15	0.19	0.40
6	γ-Caprolactone	0.03	0.04	0.03	0.02	0.03	0.03
7	(E)-Whiskey lactone	0.02	0.05	0.02	0.00	0.00	0.01
8	γ-Octalactone	0.04	0.04	0.02	0.02	0.02	0.02
9	(Z)-whiskey lactone	0.04	0.14	0.03	0.02	0.03	0.05
10	γ-Decanolactone	0.04	0.04	0.02	0.01	0.02	0.04
11	γ-Dodecalactone	0.19	0.30	0.09	0.07	0.10	0.25
	Esters	49.95	49.29	46.04	39.88	35.45	46.03
12	Ethy lacetate	0.54	1.58	0.85	0.43	0.80	1.55
13	Methyl isobutyrate	0.02	0.02	0.02	0.01	0.01	0.01
14	Ethyl propionate	0.01	0.08	0.02	0.01	0.01	0.05
15	Ethyl isobutyrate	0.00	0.01	0.00	0.00	0.00	0.01
16	Propyl acetate	0.01	0.01	0.03	0.01	0.02	0.03
17	Methyl butyrate	9.55	7.55	8.62	9.83	9.68	9.19

18	Methyl DL-2-Methylbutyrate	0.64	0.61	0.58	0.47	0.33	0.57
19	Methyl isovalerate	4.07	2.36	3.43	3.43	1.94	1.37
20	Ethyl butyrate	6.78	7.19	5.87	5.00	5.03	7.36
21	Isopropyl butyrate	4.01	4.00	2.96	2.45	1.93	2.30
22	Ethyl 2-methylbutyrate	0.18	0.55	0.19	0.13	0.10	0.61
23	Ethyl isovalerate	1.14	3.82	0.95	0.38	0.30	1.82
24	Butyl acetate	0.32	0.41	0.22	0.10	0.09	0.25
25	2-Methyl-1-butanol acetate	2.65	2.18	3.35	2.00	1.49	1.82
26	Isoamyl acetate	2.99	2.41	3.68	2.24	1.67	2.05
27	Propyl butyrate	0.20	0.43	0.21	0.10	0.11	0.23
28	Ethyl valerate	0.10	0.48	0.04	0.03	0.03	0.14
29	Amyl acetate	0.19	0.21	0.29	0.37	0.17	0.28
30	Methyl hexanoate	7.70	6.02	7.05	8.02	8.24	7.38
31	Ethyl hexanoate	4.62	5.41	3.82	2.22	1.53	5.61
32	Isoamyl butyrate	1.36	0.45	0.24	0.10	0.07	0.18
33	Hexyl acetate	0.44	0.35	0.58	0.71	0.37	0.80
34	Amyl butyrate	0.00	0.10	0.00	0.00	0.00	0.00
35	cis-3-Hexenyl acetate	0.33	0.24	0.50	0.57	0.32	0.41
36	Propyl hexanoate	0.01	0.03	0.01	0.00	0.00	0.01
37	2-Ethyl hexyl acetate	0.04	0.06	0.02	0.02	0.00	0.02
38	Methyl octanoate	0.74	0.51	1.07	0.38	0.51	0.47
39	Methyl(methylthio)acetate	0.01	0.00	0.01	0.01	0.00	0.02
40	Butyl hexanoate	0.04	0.08	0.01	0.00	0.00	0.02
41	Ethyl octanoate	0.11	0.48	0.14	0.04	0.04	0.31
42	Isoamyl hexanoate	0.06	0.11	0.04	0.02	0.01	0.03
43	Nonyl acetate	0.00	0.01	0.00	0.00	0.00	0.01

44	Hexyl hexanoate	0.04	0.05	0.04	0.04	0.02	0.06
45	Methyl benzoate	0.23	0.17	0.24	0.33	0.25	0.21
46	Ethyl decanoate	0.00	0.04	0.00	0.00	0.00	0.02
47	Isopropyl benzoate	0.03	0.02	0.03	0.03	0.02	0.02
48	Ethyl benzoate	0.06	0.07	0.06	0.04	0.03	0.09
49	Benzyl acetate	0.07	0.07	0.08	0.03	0.02	0.11
50	Birch-Me	0.36	0.71	0.50	0.19	0.18	0.24
51	(2Z)-3,7-dimethyl-2,6-octadien-1-yl butyrate	0.00	0.01	0.02	0.03	0.02	0.02
52	Methyl cinnamate	0.06	0.05	0.03	0.04	0.04	0.05
53	Ethyl cinnamate	0.11	0.16	0.03	0.05	0.03	0.10
54	Methyl anthranilate	0.13	0.22	0.22	0.06	0.05	0.20
	Aldehydes	22.93	18.78	17.23	25.86	26.87	22.78
55	Butyraldehyde	0.05	0.03	0.04	0.07	0.07	0.05
56	Caproaldehyde	8.41	6.87	7.33	10.37	10.50	8.75
57	trans-2-Pentenal	0.43	0.33	0.22	0.71	0.65	0.43
58	trans-2-Hexen-1-al	8.24	6.23	6.59	9.56	9.78	7.64
59	Octanal	0.19	0.16	0.08	0.15	0.20	0.17
60	trans-2-Heptenal	1.19	0.85	0.54	1.16	1.58	1.09
61	Melonal	0.00	0.00	0.07	0.00	0.00	0.00
62	Nonanal	0.41	0.30	0.22	0.28	0.33	0.31
63	2,4-Hexadienal	0.08	0.06	0.05	0.06	0.08	0.07
64	(E)-2-Octenal	0.88	0.72	0.44	0.85	0.92	1.00
65	Benzaldehyde	2.21	2.42	0.92	1.87	1.79	2.21
66	trans-Cinnamaldehyde	0.08	0.07	0.19	0.09	0.12	0.08
67	trans-2-Nonenal	0.14	0.13	0.15	0.10	0.13	0.21
68	trans,trans-2,4-Heptandienal	0.12	0.12	0.08	0.18	0.19	0.14

69	trans,cis-2,6-Nonadienal	0.15	0.13	0.11	0.14	0.15	0.19
70	(Z)-2-Decenal	0.09	0.05	0.00	0.00	0.09	0.06
71	trans-2-Decenal	0.02	0.01	0.01	0.01	0.02	0.01
72	trans,trans-2,4-Nonadienal	0.04	0.04	0.03	0.04	0.05	0.05
73	trans-2,4-Decadienal	0.12	0.15	0.13	0.19	0.18	0.22
74	Pentadecanal	0.06	0.09	0.03	0.02	0.03	0.11
75	Decanal	0.02	0.02	0.01	0.02	0.02	0.02
	Ketones	4.84	5.82	5.09	5.00	4.87	4.47
76	2-Butanone	0.06	0.07	0.07	0.06	0.06	0.08
77	3-Methyl-2-butanone	0.01	0.01	0.02	0.02	0.01	0.01
78	2-Pentanone	0.37	0.75	0.00	0.00	0.00	0.41
79	3-Pentanone	0.00	0.00	0.89	0.65	0.19	0.00
80	2,3-Butanedione	0.82	0.80	1.14	0.62	0.56	0.44
81	2-Methyl-3-pentanone	0.00	0.00	0.00	0.00	0.00	0.00
82	4-Methyl-2-pentanone	0.32	0.57	0.35	0.22	0.13	0.29
83	Ethyl vinyl ketone	0.98	0.70	0.44	1.49	1.36	1.06
84	2-Heptanone	0.89	1.85	1.00	0.49	0.47	0.80
85	3-Octanone	0.17	0.16	0.24	0.25	0.26	0.21
86	2-Octanone	0.00	0.01	0.01	0.01	0.01	0.01
87	1-Octen-3-one	0.63	0.38	0.13	0.48	0.86	0.58
88	Methyl heptenone	0.48	0.37	0.38	0.55	0.70	0.46
89	3-Octen-2-one	0.01	0.02	0.00	0.02	0.03	0.02
90	2-Methyl tetrahydrothiophen-3-one	0.01	0.01	0.01	0.01	0.01	0.01
91	Acetophenone	0.09	0.11	0.42	0.12	0.20	0.10
	Alcohols	3.25	2.53	14.34	11.44	11.55	4.61
92	(±)-2-Pentanol	0.01	0.01	0.00	0.00	0.00	0.00

93	n-Butanol	0.13	0.10	0.07	0.11	0.06	0.12
94	(±)-2-Methyl-1-butanol	0.09	0.08	0.09	0.10	0.12	0.11
95	Isopentyl alcohol	0.41	0.28	0.33	0.52	0.50	0.32
96	1-Pentanol	0.64	0.43	1.36	1.03	1.27	0.70
97	2-Heptanol	0.10	0.15	0.06	0.04	0.03	0.07
98	1-Hexanol	0.94	0.77	6.36	5.32	5.78	2.18
99	trans-3-Hexen-1-ol	0.01	0.00	0.13	0.06	0.05	0.02
100	Leaf alcohol	0.03	0.02	0.08	0.11	0.07	0.04
101	trans-2-Hexen-1-ol	0.36	0.23	4.49	3.44	2.72	0.49
102	cis-2-Hexen-1-ol	0.00	0.00	0.03	0.00	0.00	0.00
103	1-Octen-3-ol	0.15	0.10	0.20	0.17	0.24	0.15
104	1-Heptanol	0.11	0.08	0.29	0.12	0.17	0.10
105	2-Ethyl-1-hexanol	0.07	0.06	0.06	0.08	0.08	0.07
106	n-Octanol	0.11	0.10	0.34	0.15	0.18	0.10
107	trans-2-Octen-1-ol	0.00	0.02	0.05	0.04	0.04	0.03
108	1-Nonanol	0.07	0.07	0.14	0.12	0.13	0.09
109	Benzyl alcohol	0.03	0.03	0.08	0.04	0.05	0.04
110	Chnnamyl alcohol	0.00	0.00	0.18	0.00	0.06	0.00
	Acids	11.09	14.85	10.26	10.26	14.21	11.87
111	Isobutyric acid	0.42	0.74	0.39	0.36	0.51	0.39
112	Butyric acid	1.07	2.59	0.90	0.64	1.01	0.93
113	2-Methylbutyric acid	1.32	2.65	1.41	1.23	1.49	1.70
114	Valeric acid	0.20	0.22	0.15	0.21	0.46	0.29
115	Hexanoic acid	7.03	7.16	6.14	6.80	8.51	6.73
116	5-Methylhexanoic acid	0.00	0.03	0.00	0.00	0.00	0.00
117	trans-2-Hexenoic acid	0.06	0.06	0.09	0.10	0.14	0.08

118	n-Octanoic acid	0.23	0.51	0.29	0.17	0.43	0.40
119	Nonoic acid	0.73	0.85	0.83	0.71	1.59	1.29
120	Capric acid	0.04	0.05	0.05	0.04	0.07	0.06
	Terpenoids	5.41	4.91	4.23	4.23	4.34	6.34
121	Camphene	0.00	0.00	0.00	0.01	0.01	0.00
122	3-Carene	0.00	0.00	0.00	0.00	0.00	0.00
123	Myrcene	0.05	0.00	0.05	0.05	0.05	0.06
124	Cinene	0.01	0.01	0.01	0.01	0.02	0.01
125	Eucalyptol	0.00	0.00	0.00	0.00	0.00	0.00
126	(Z)-β-ocimene	0.02	0.02	0.02	0.02	0.02	0.02
127	trans-β-Ocimene	0.02	0.02	0.03	0.02	0.02	0.03
128	Terpinolene	0.01	0.01	0.01	0.01	0.01	0.01
129	(±)-Camphor	0.02	0.01	0.02	0.03	0.03	0.03
130	Linalool	3.83	3.06	3.30	3.40	3.23	3.79
131	trans-β-Farnesene	0.06	0.08	0.03	0.02	0.04	0.12
132	Linalyl butyrate	0.02	0.07	0.00	0.00	0.00	0.02
133	alpha-Terpineol	0.01	0.02	0.02	0.02	0.01	0.01
134	β-Citronellol	0.04	0.03	0.02	0.03	0.04	0.03
135	Geranyl acetone	0.05	0.03	0.03	0.06	0.07	0.05
136	β-Ionone	0.02	0.02	0.02	0.04	0.04	0.03
137	Nerolidol	0.99	1.39	0.49	0.32	0.54	1.94
138	α-Pinene	0.00	0.00	0.00	0.00	0.00	0.00
139	cis-Linalool oxide	0.05	0.02	0.04	0.04	0.04	0.03
140	(E)-Linalool oxide	0.14	0.07	0.09	0.09	0.13	0.10
141	Methyl disulfide	0.01	0.01	0.03	0.02	0.03	0.02
	Others	0.13	0.11	0.16	0.15	0.38	0.33

142	Phellandrene	0.06	0.05	0.05	0.05	0.05	0.06
143	Pyridine	0.00	0.00	0.00	0.00	0.20	0.21
144	2-Ethyl pyridine	0.01	0.01	0.01	0.01	0.01	0.01
145	Dimethyl trisulfide	0.01	0.01	0.03	0.02	0.03	0.01
146	4-Isopropenyltoluene	0.06	0.05	0.06	0.07	0.08	0.06
147	Benzothiazole	0.03	0.03	0.03	0.03	0.04	0.03

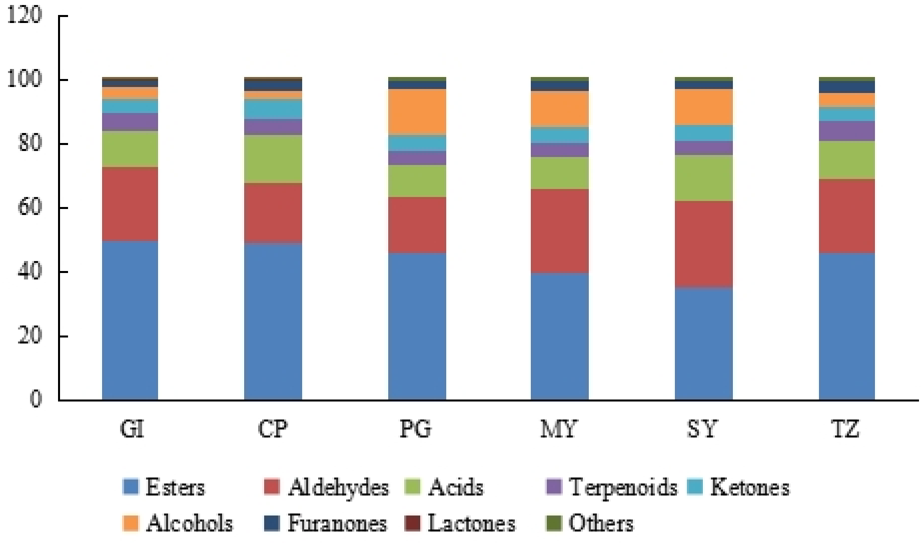

Figure S1. Schematic diagram representing the volatile compound abundance in the major chemical classes detected in strawberry fruits from different groups

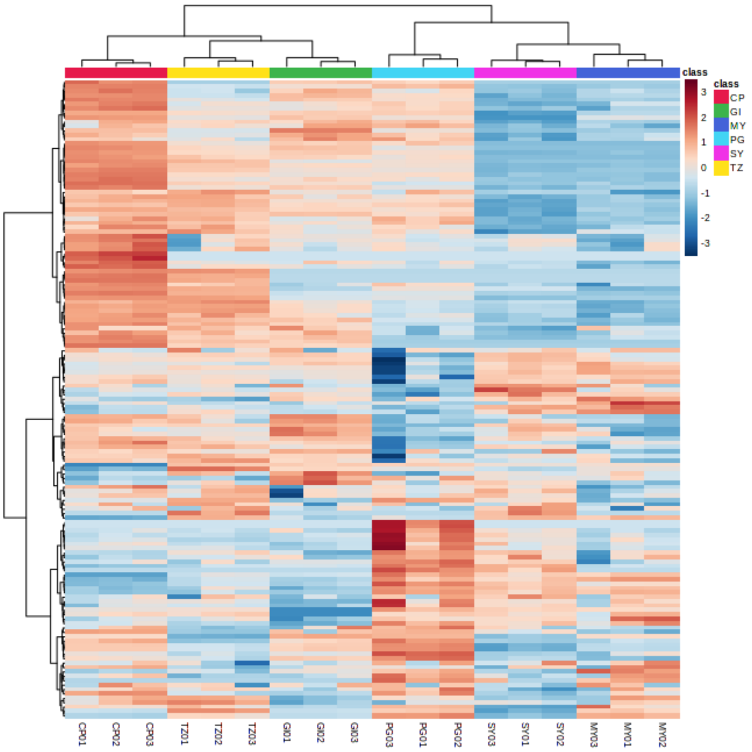

Figure S2. Cluster heat map of strawberry samples on the basis of volatile compounds

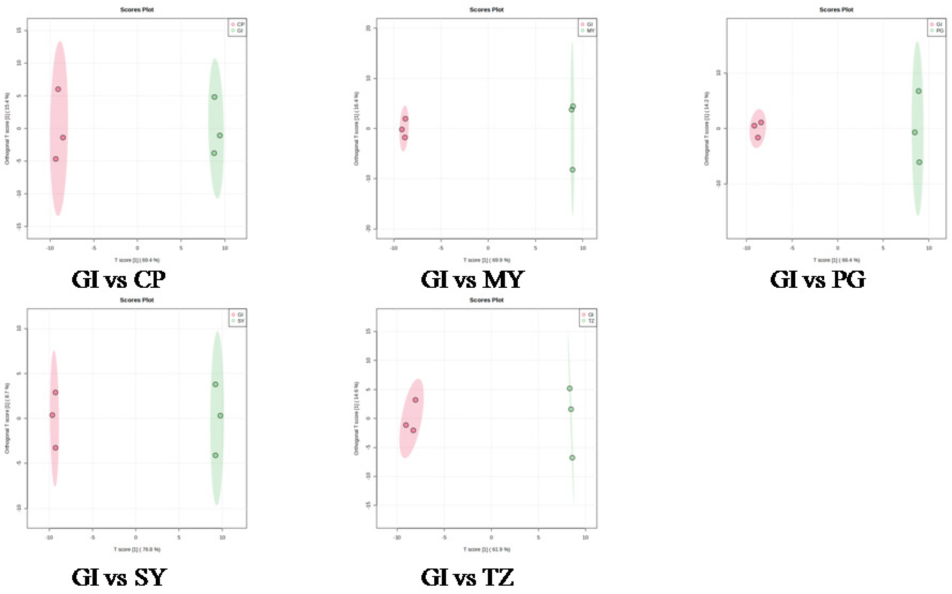

Figure S3. OPLS-DA score plots of strawberry samples between GI group and other groups on the basis of volatile compounds

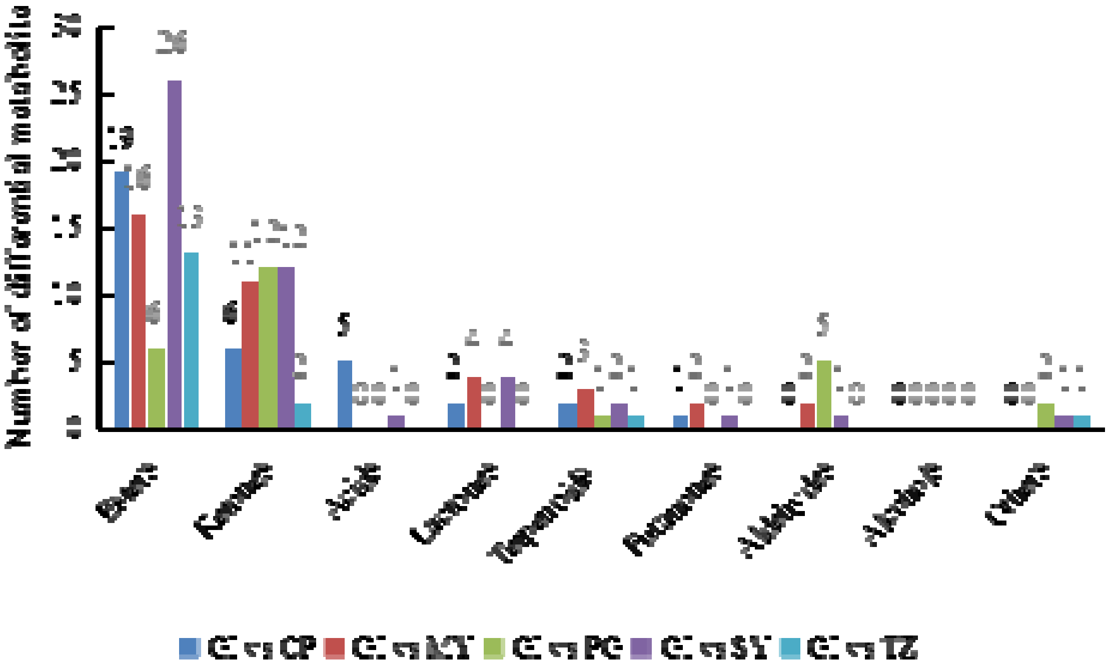

Figure S4. Differential volatile compounds in the major chemical classes detected in strawberry samples from different groups

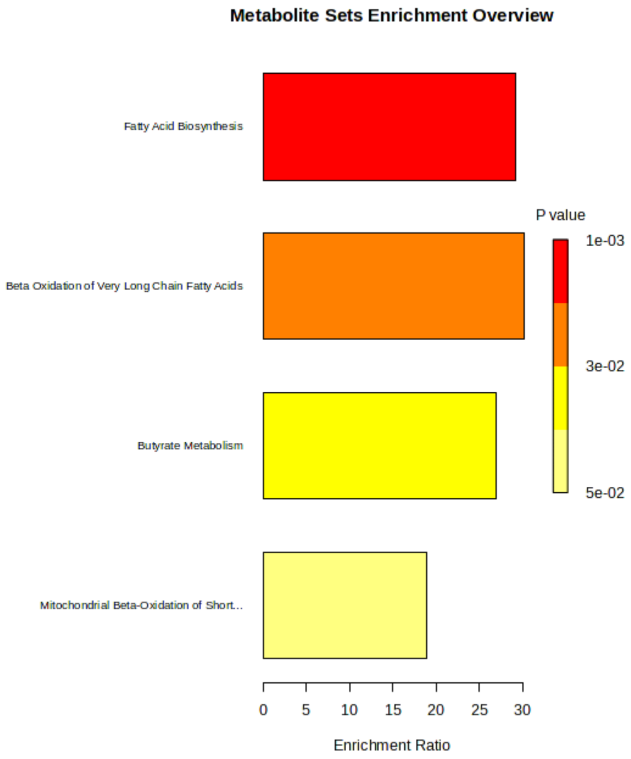

Figure S5. KEGG barplots of GI versus CP

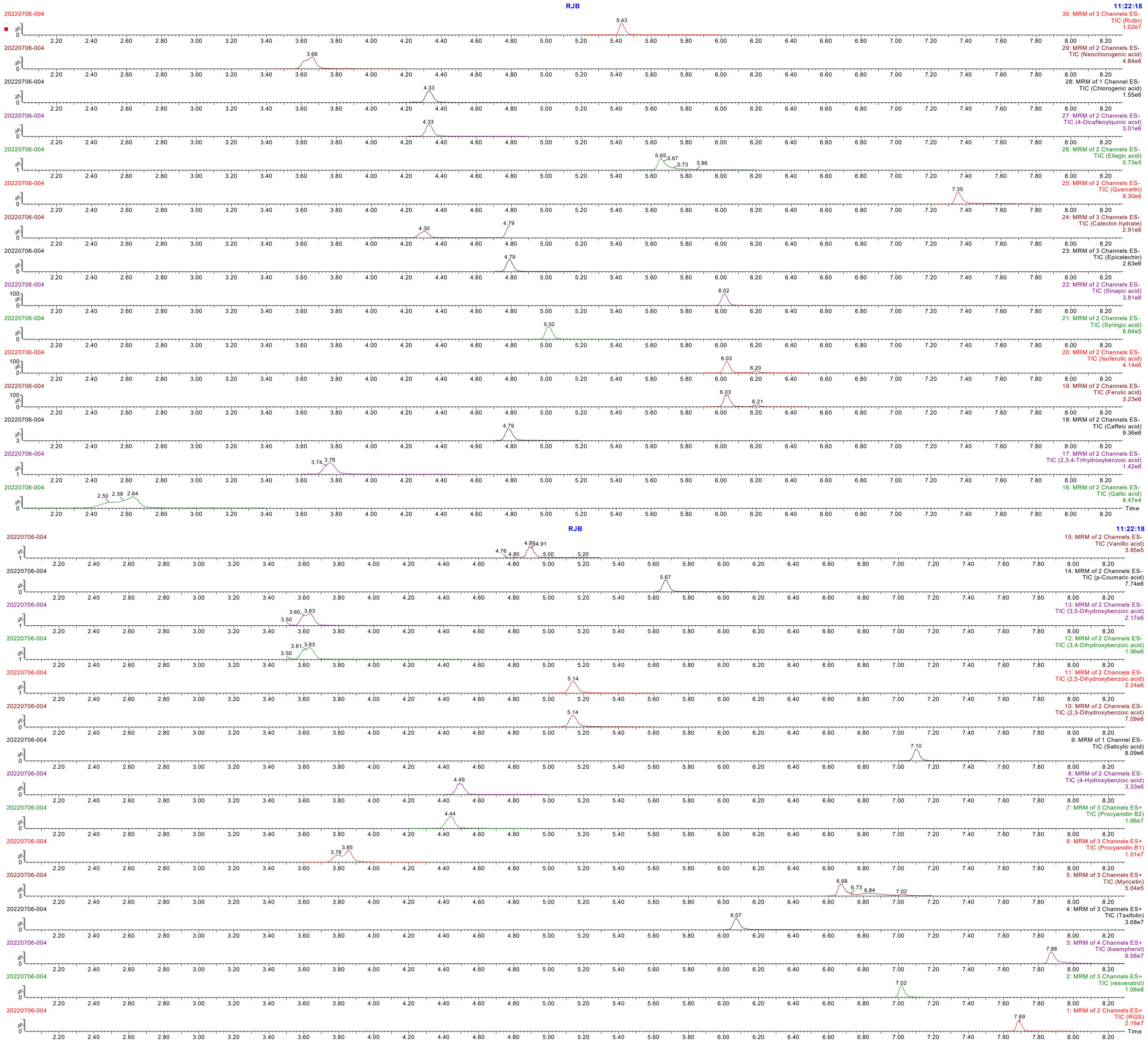

Figure S6. Chromatogramsof 30 kinds of phenolic acids

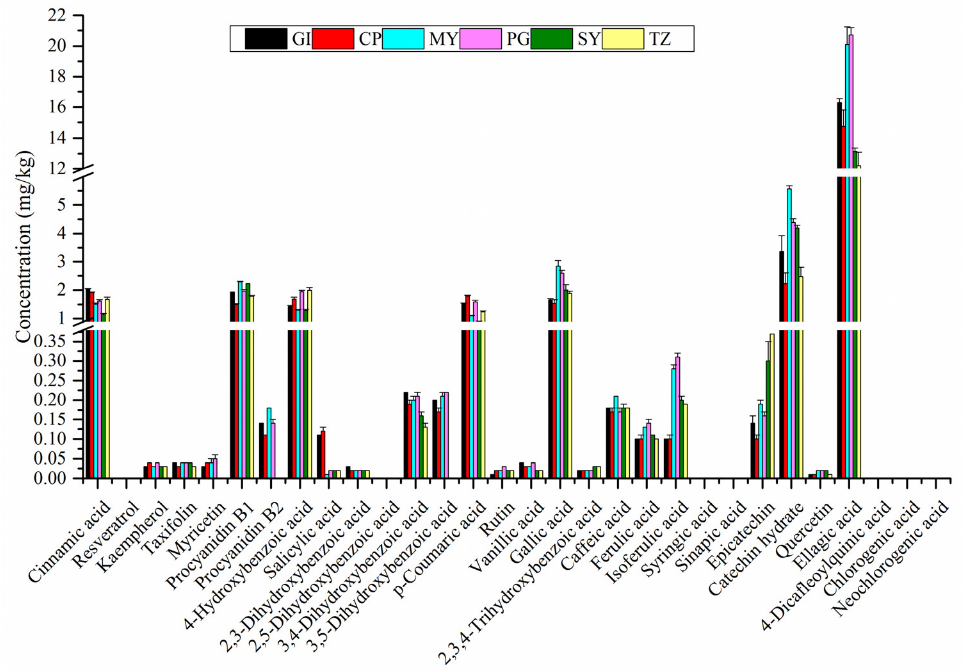

Figure S7. Contents of phenolic acids (mg/kg FW ± RSD (n = 3)) in strawberry samples from six groups

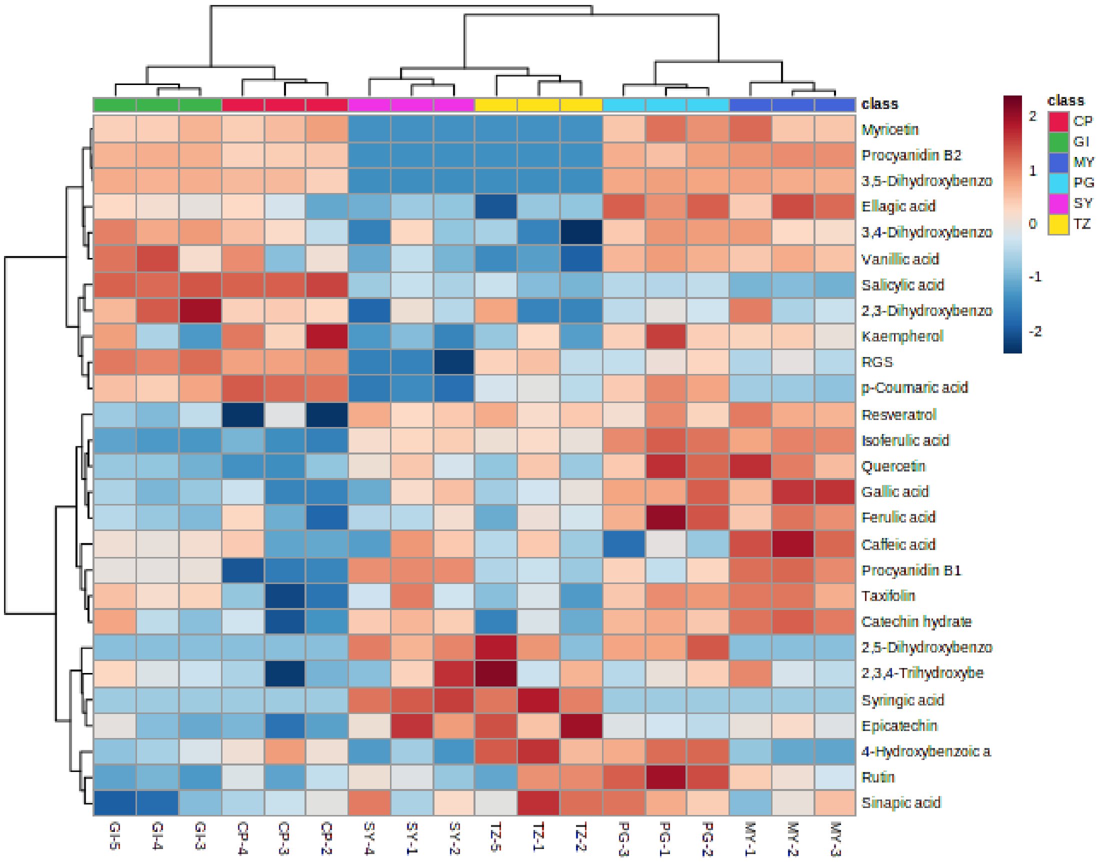

Figure S8. Cluster heat map of strawberry samples on the basis of phenolic acids

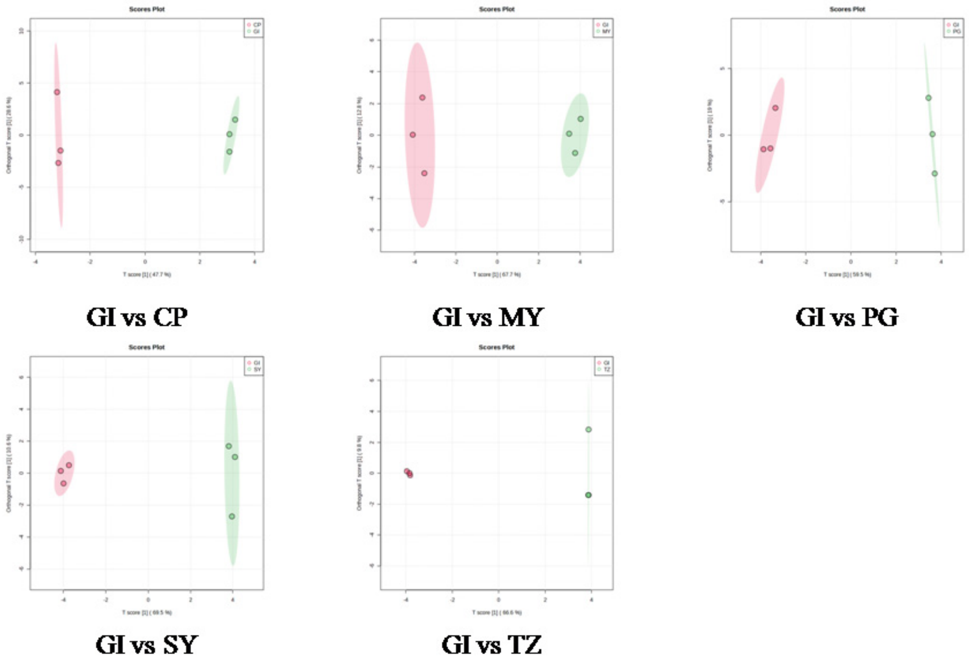

Figure S9. OPLS-DA score plots of strawberry samples between GI group and other groups on the basis of phenolic acids
